# The interrelationship of proteasome impairment and oligomeric intermediates in neurodegeneration

**DOI:** 10.1111/acel.12359

**Published:** 2015-06-05

**Authors:** Jennifer M Deger, Julia E Gerson, Rakez Kayed

**Affiliations:** Departments of Neurology, Neuroscience and Cell Biology, Mitchell Center for Neurodegenerative Diseases, University of Texas Medical Branch301 University Building, Medical Research Building, Galveston, TX, 77555-1045, USA

**Keywords:** Alzheimer’s disease, covalent modification, Huntington’s disease, neurodegeneration, Parkinson’s disease, small ubiquitin-like modifiers, tau oligomers, tauopathies, ubiquitin proteasome system, ubiquitination

## Abstract

Various neurodegenerative diseases are characterized by the accumulation of amyloidogenic proteins such as tau, α-synuclein, and amyloid-β. Prior to the formation of these stable aggregates, intermediate species of the respective proteins—oligomers—appear. Recently acquired data have shown that oligomers may be the most toxic and pathologically significant to neurodegenerative diseases such as Alzheimer’s and Parkinson’s. The covalent modification of these oligomers may be critically important for biological processes in disease. Ubiquitin and small ubiquitin-like modifiers are the commonly used tags for degradation. While the modification of large amyloid aggregates by ubiquitination is well established, very little is known about the role ubiquitin may play in oligomer processing and the importance of the more recently discovered sumoylation. Many proteins involved in neurodegeneration have been found to be sumoylated, notably tau protein in brains afflicted with Alzheimer’s. This evidence suggests that while the cell may not have difficulty recognizing dangerous proteins, in brains afflicted with neurodegenerative disease, the proteasome may be unable to properly digest the tagged proteins. This would allow toxic aggregates to develop, leading to even more proteasome impairment in a snowball effect that could explain the exponential progression in most neurodegenerative diseases. A better understanding of the covalent modifications of oligomers could have a huge impact on the development of therapeutics for neurodegenerative diseases. This review will focus on the proteolysis of tau and other amyloidogenic proteins induced by covalent modification, and recent findings suggesting a relationship between tau oligomers and sumoylation.

## Introduction

Proteostasis is a critical process thought to go awry in neurodegenerative diseases and other disorders characterized by the build-up of toxic proteins. Proteolysis may occur by autophagic degradation in the lysosome or by targeted breakdown in the proteasome. While autophagy was commonly viewed as a nonselective process when compared to the proteasome, recent studies have shown that it may be more selective than previously thought and its dysfunction in neurodegenerative disease has been well established (Reggiori *et al*., 2012; Vidal *et al*., 2014). On the other hand, proteasomal degradation in eukaryotic cells is primarily controlled by the peptide ubiquitin. Ubiquitin tags the targeted protein in a covalent bonding process called conjugation, and as a consequence, the protein is sent to the proteasome for digestion (Hochstrasser, 1996). The degraded protein component amino acids are then often reused by the cell. The ubiquitin proteasome system (UPS) has long been a subject of extensive biomedical research because of its importance to proper cellular function. A better understanding of the UPS has the potential to help patients afflicted with cancer, inflammatory, autoimmune, and neurodegenerative diseases, to name a few (Delobel *et al*., 2005; Wang & Maldonado, 2006).

With the increasing age of the general population, neurodegenerative diseases are becoming more prevalent and taxing to the healthcare system. The UPS appears to have numerous implications for neurodegeneration and is thus an important research topic. Neural cells in patients plagued with Parkinson’s disease (PD), Huntington’s disease (HD), Alzheimer’s disease (AD), amyotrophic lateral sclerosis (ALS), and other diseases of the nervous system (Table1) have characteristics that suggest a faulty UPS could be one of the first significant links in the chain of events leading to neurotoxicity (Olanow & McNaught, 2006). One prominent example is the correlation between neurodegeneration that runs in families and mutations in genes encoding important components of the UPS (Leroy *et al*., 1998). The inhibition of proteasome activity allows for defective proteins to build up to toxic levels.

**Table 1:** Summary of diseases and amyloid proteins described

Disease	Proteins implicated	
Parkinson’s disease (PD)	α-synuclein	Tau
Alzheimer’s disease (AD)	Amyloid-β	Tau
Huntington’s disease (HD)	Huntingtin	Tau
Amyotrophic lateral sclerosis (ALS)	TDP43	Tau
Frontotemporal lobar dementia	Tau	
Traumatic brain injury	Tau	
Scrapie	Prion (PrP(Sc))	

Ubiquitination is not the only covalent modification implicated in proteolysis and neurodegeneration. Recently, a family of ubiquitin-like proteins was discovered, including small ubiquitin-like modifiers (SUMO). Sumoylation, the covalent attachment of SUMO to a targeted protein, provides a reversible, rapid, and efficient way to regulate biological processes (Gill, 2004). Many proteins involved in neurodegeneration are found to be sumoylated, including tau (Dorval & Fraser, 2006). In AD, defective tau proteins form intracellular neurofibrillary tangles (NFTs), and misfolded amyloid-β (Aβ) proteins form extracellular senile plaques. These insoluble, toxic inclusions are considered the final faulty forms of their respective proteins and were widely accepted as the toxic species in neurodegenerative disorders. However, recent evidence suggests that these proteins do not have to progress to these late stages in the aggregation pathway to cause neurotoxicity. Soluble intermediate forms called oligomers may be the most toxic species present before the more mature filaments develop. Tau oligomers, in particular, are emerging as a novel target for disease intervention (Gerson *et al*., 2014b).

Post-translational modifications of tau oligomers such as phosphorylation, polyubiquitination, and truncation can alter tau’s biological function and self-assembly (Morishima-Kawashima *et al*., 1995). The covalent attachment of ubiquitin or SUMO to tau oligomers may also have important implications for proper cell functioning, and the investigation of such phenomena is the topic of many current research projects. In a fully operational proteolytic pathway, potentially harmful oligomers can be tagged and destroyed before inducing a cascade of toxic effects. Many studies report a high rate of ubiquitination and sumoylation in brains afflicted with neurodegenerative disorders (Alves-Rodrigues *et al*., 1998; Lasagna-Reeves *et al*., 2012a; Tai *et al*., 2012). Therefore, it is logical to conclude that while diseased cells may not have difficulty in recognizing and tagging defective proteins, problems may arise at the proteasome, where degradation is supposed to take place.

Research over the past few years has provided evidence that the ubiquitination and sumoylation systems often communicate with one another and jointly affect the properties of common substrate proteins, in some cases by targeting them to the same site and in others by acting antagonistically or even sequentially (Desterro *et al*., 2005; Ulrich, 2005). The obscure relationship between the two systems and their effects on tau oligomers may play a crucial role in neurodegeneration and is currently a topic of great research interest. The post-translational modification of tau oligomers, whether by ubiquitination or sumoylation, has important implications in the development and progression of neurodegenerative diseases. This review will summarize the knowledge of covalent modification of tau and other amyloidogenic proteins leading to proteolysis and discuss the sumoylation of tau oligomers, as it has yet to be investigated thoroughly and has important implications for understanding neurodegeneration.

## The UPS

The UPS appears to be impaired in neurodegenerative disease. This malfunction may occur in one of two ways: It could become overactive and needlessly destroy useful proteins, or it could become overly restrained and allow harmful proteins to build up to toxic levels, as appears to be the case in tauopathies such as AD (Song & Jung, 2004).

### Ubiquitination

Ubiquitin is a small 76 amino acid protein crucial to the selective degradation of various cytosolic, nuclear, and endoplasmic reticulum proteins (Hochstrasser, 1996). Degradation is enhanced when more than one ubiquitin is attached to the protein to form a polyubiquitin chain (Cook *et al*., 1994). Various types of neurodegeneration are characterized by intraneuronal inclusions comprised of ubiquitinated proteins. Although the precise methods contributing to the formation of these abnormal protein deposits are obscure, the involvement of ubiquitin in neurodegeneration is so widely accepted that ubiquitin immunoreactivity is used regularly to identify the Lewy bodies and NFTs associated with neurological disorders (Alves-Rodrigues *et al*., 1998). Moreover, increased levels of free ubiquitin pools have been observed in AD (Taddei *et al*., 1993), PD (Sugiyama *et al*., 1994), and ALS (Schiffer *et al*., 1994), when compared to controls.

Further, supporting the importance of ubiquitination is a study on mouse brains infected with scrapie. In these brains, ubiquitinated proteins were identified long before clinical signs of the disease appeared (Mayer *et al*., 1996). This demonstrates that ubiquitin is able to recognize pathological prion protein (PrP(Sc)) in the early stages of scrapie and suggests that the same may be true for other neurodegenerative diseases affected by prion-like amyloid proteins.

It is likely that defective proteins are somehow rendered unattainable to the proteasome, even though the cell is able to recognize the defect and tag it for destruction via ubiquitination. Failure to eliminate these ubiquitinated proteins may lead to the intracellular and extracellular inclusions hallmarked as the neurotoxic species in neurodegenerative diseases. However, the mechanism for this malfunction is unknown. In theory, if the UPS was fully functional, neurotoxicity could be avoided altogether.

### Sumoylation

A SUMO, similar in structure to ubiquitin, has a multitude of diverse functions important for the majority of cellular pathways, including the regulation of signal transduction pathways, transcription, nucleocytoplasmic transport, the cell cycle, and chromosome integrity and genomic stability (Melchior, 2000; Gill, 2004; Johnson, 2004; Hay, 2005).

Three major genetic paralogs of SUMO are expressed in humans: SUMO-1, SUMO-2, and SUMO-3. A gene encoding SUMO-4 has been identified, but any expression of this gene has yet to be discovered and it is thought to be an evolutionary artifact (Bohren *et al*., 2007). Of the three expressed types of SUMO, SUMO-2 and SUMO-3 are most similar to one another (Sarge & Park-Sarge, 2009). The three paralogs have unique subcellular localization patterns (Zhang *et al*., 2008), and SUMO-1 responds differently to heat shock and stress due to its less dynamic nature (Ayaydin & Dasso, 2004; Wang & Dasso, 2009).

While many SUMO targets are conjugated to all forms of SUMO, some are specifically conjugated to only one paralog (Vertegaal *et al*., 2006), including some proteins that are implicated in neurodegeneration. The pool of free SUMO-2 and SUMO-3 available for conjugation is notably higher than that of SUMO-1 due to the higher overall cellular concentration, suggesting that SUMO-2 and SUMO-3 perform the majority of sumoylation (Saitoh & Hinchey, 2000). However, both tau and α-synuclein are preferentially modified by SUMO-1, as opposed to SUMO-2 or SUMO-3(Takahashi *et al*., 2008). It is possible that these differences may underlie an altered mechanism of degradation for these proteins that may be either more or less efficient than the more common sumoylation pathways.

The addition of SUMO to components of the transcriptional apparatus does not have a common consequence as it can both activate and repress transcription (Girdwood *et al*., 2004). A unique feature of SUMO is that only a small fraction of its substrate is sumoylated at any given time (Johnson, 2004). Although the exact purpose of sumoylation and its specific targets are still unclear, available data provide compelling evidence for a role of SUMO in the regulation of protein–protein interactions and in subcellular localization (Melchior, 2000). Small ubiquitin-like modifier has implications in the proteolytic pathways of the cell and therefore is an area of great research interest for many diseases, including neurodegenerative disorders.

### The complex relationship between ubiquitination and sumoylation

As both sumoylation and ubiquitination are critical components of cellular metabolism, it is unsurprising that the two systems would affect one another. Both systems are reversible, and in some cases, dynamic cycles of modification may be required for activity (Gill, 2004). Many proteins are substrates for both ubiquitin and SUMO; however, the two tags at times seem to have an antagonistic relationship, targeting the same proteins to different fates—with SUMO frequently acting to repress transcription while ubiquitin often increases gene expression (Gill, 2004; Desterro *et al*., 2005). Some studies have demonstrated that SUMO acts by blocking ubiquitin attachment sites (Johnson, 2004). Specifically, one project reported that the phosphorylated tau in AD is ubiquitinated in regions that contain sumoylation sites (Morishima-Kawashima *et al*., 1995).

Despite the information provided by recent studies on the subject, the interactions between ubiquitination and sumoylation are highly complex and require further investigation to be better understood. Some research suggests that the two act successively, while some suggest that they act independently of one another. The two may jointly exert regulatory control over biological processes where the identity of the targets may at first glance point to an antagonistic relationship (Ulrich, 2005).

## Inhibition of the UPS corresponds to protein aggregation and cytotoxicity

It is clear that dysfunction in the UPS may lead to the accumulation of toxic protein aggregates in a multitude of neurodegenerative disorders. However, it is unknown whether protein accumulation harms the function of the UPS or whether the failure of the UPS leads to protein accumulation. Sifting out causation verses correlation is one of the first steps to understanding these complex cellular systems. However, the majority of research supports the latter theory that a faulty UPS allows proteins to accumulate. Mutations in the genes encoding components of the UPS are linked to neurodegeneration, suggesting that protein aggregation is a result of these abnormalities and not the initial cause of toxicity. Importantly, once aggregation begins, it may then further impair the UPS, acting as a positive feedback mechanism (Bence *et al*., 2001). As more protein accumulates, it inhibits the UPS to a greater extent and begins to accumulate at an increasing rate. The exacerbating effect of neurotoxicity on the UPS would explain why neurodegeneration tends to progress exponentially. Specific examples supporting the claim that UPS impairment leads to neurotoxicity will be discussed in greater detail in relation to particular neurodegenerative diseases such as PD, HD, and AD.

### Proteasome impairment and α-synuclein

Synucleins are a family of proteins, abundant in the brain, whose specific biological function is relatively unclear (Jakes *et al*., 1994), although evidence supports a potential role in membrane plasticity (Clayton & George, 1999). Synucleins are believed to be natively unfolded proteins with little consistency in secondary structure formation, and their filamentary forms are thought to be polar (Serpell *et al*., 2000; Goedert *et al*., 2001; Dunker *et al*., 2008). α-synuclein is widely recognized as a molecular hallmark of several neurodegenerative conditions now designated as synucleinopathies (Fernagut & Chesselet, 2004). One unique feature of α-synuclein is its ability to readily form aggregates *in vitro* without the need of other co-factors (Giasson *et al*., 2003). Evidence supporting the theory that aggregations of α-synuclein are toxic includes a transgenic mouse model in which overexpression of human α-synuclein is correlated with neurodegeneration and locomotive deficits (Fernagut & Chesselet, 2004).

α-Synuclein is a major component of Lewy bodies and Lewy neurites, the inclusions that mark PD (Spillantini *et al*., 1998; Goedert *et al*., 2001) and Lewy body dementia (LBD). Parkinson’s disease is characterized by the accumulation of proteins in dopaminergic neurons, leading to cell death. Toxic α-synuclein oligomers go on to form large, stable aggregates called Lewy bodies and Lewy neurites in PD that are frequently ubiquitinated (Spillantini *et al*., 1998; Goedert *et al*., 2001; Shimura *et al*., 2001). Importantly, α-synuclein appears to interact with ubiquitin prior to the formation of fibrillar aggregates. In a cellular model, ubiquitin levels were shown to positively correlate with levels of α-synuclein oligomers, an increase that corresponded with UPS dysfunction (Martins-Branco *et al*., 2012). Parkinson’s disease affects a large portion of the population and can be either sporadic or familial. It causes locomotive impairment, cognitive deficits, and shortened life expectancy (Lang & Lozano, 1998). Lewy body dementia, common late in life and often overlapping with characteristics of AD such as Aβ deposits, is also marked by Lewy bodies and Lewy neurites (Goedert *et al*., 2001). α-synuclein has appeared in Lewy body pathology associated with sporadic and familial AD, Down’s syndrome, multiple system atrophy, and in a small fraction of Hallervorden-Spatz cases (Lippa *et al*., 1998; Tu *et al*., 1998). α-synuclein has implications for a multitude of major neurodegenerative diseases and is therefore at the center of intensive research.

One of the most promising experimental models for PD is a transgenic fly, *Drosophila melanogaster*, whose nerve cells express wild-type or mutant human α-synuclein, leading to the formation of filamentous inclusions that resemble Lewy bodies (Feany & Bender, 2000). Although the data from this model have provided no evidence that a synuclein homolog exists (Rubin *et al*., 2000), these flies express human α-synuclein more readily than transgenic mice, suggesting that vertebrates or possibly mammals have developed mechanisms that prevent α-synuclein assembly. Such mechanisms have many implications in the progression of α-synucleinopathies (Goedert *et al*., 2001).

Both *in vitro* and *in vivo* laboratory experiments have provided evidence that the inhibition of the UPS may result in protein aggregation and cytotoxicity in PD (Olanow & McNaught, 2006). A defective UPS could very well be the cause of such neurodegeneration, albeit indirectly. Unwanted proteins are allowed to mature and accumulate because they are not properly digested, ultimately leading to neurodegeneration. Ideally, a functional UPS would degrade the unwanted proteins into their component amino acids and prevent accumulation from happening in the first place.

Genetic evidence suggests that toxicity may be a result of UPS impairment. Several familial forms of PD are characterized by genetic mutations that inhibit the proper formation of the protein α-synuclein and two enzymes of the UPS, parkin and ubiquitin C-terminal hydrolase L1 (UCH-L1). In one German family with PD, the mutation Ile93Met hinders the catalytic activity of UCH-L1, thus causing irregularity in the UPS and allowing protein accumulation (Leroy *et al*., 1998). Levels of ubiquitin expression are controlled by the balance of different types of enzymes: ubiquitin activating, ubiquitin conjugating, ubiquitin ligases, and deubiquitinating enzymes. UCH-L1 is a deubiquitinating enzyme and a constituent of the Lewy bodies that has been proven to play a critical role in the ubiquitin-dependent proteolytic pathway (Harada *et al*., 2004). Once the targeted protein binds to the proteasome, the ubiquitin chain is supposed to be removed so that the next steps in the degradation process may take place. Faulty UCH-L1 cannot effectively cleave the covalent bond between ubiquitin and substrates, and therefore, the amount of free ubiquitin in the brain decreases. The depletion of free-floating ubiquitin could allow misfolded proteins to aggregate, appearing to contradict studies that have shown an increase in the levels of free ubiquitin pools in PD (Sugiyama *et al*., 1994). However, studies on ubiquitin homeostasis suggest that both the overexpression and the loss of ubiquitin can lead to similar neurodegenerative phenotypes, thereby indicating that an increased UPS response overcompensating for a large increase in the level of protein aggregates in the disease may actually lead to increased toxicity, rather than simply depletion of toxic aggregates (Chen *et al*., 2009, 2011; Hallengren *et al*., 2013). Moreover, these results indicate that proper ubiquitin homeostasis is of great importance in neurodegeneration; while the levels cannot be depleted, they also must not be too high. These conditions may alter during the disease, with free ubiquitin initially peaking and then later depleting as the UPS becomes increasingly taxed by the influx of protein aggregates it is targeting for degradation. However, the mutation of UCH-L1 is controversial as some studies have failed to find the same genetic mutation in other families with autosomal dominant PD and thus concluded that the Ile93Met mutation in UCH-L1 gene is a very rare cause of familial PD (Zhang *et al*., 2000). Despite these suppositions, there is no doubt that the few cases of such familial mutations demonstrate a clear example of a relationship between the UPS and PD.

Although investigation is needed to fully understand the role of UCH-L1 in the proteolytic system, the available evidence strongly suggests the UPS as one of the causes of PD. Even if UCH-L1’s mutation is not at the root of PD, other components of the cell’s proteolytic pathway have shown abnormalities in brains afflicted with the disease. Parkin is another ubiquitinating enzyme, specifically an E3 ubiquitin ligase, whose mutations are associated with inherited forms of PD. Research has demonstrated that α-synuclein is a substrate for parkin’s ubiquitin ligase activity in normal human brains and that the loss of parkin function causes pathological protein accumulation (Shimura *et al*., 2001). Additionally, reduced levels of the proteasome activators PA700 and PA28 have been observed in the substantia nigra pars compacta (SNc) of patients with PD, both those who were genetically predisposed to the disease and those who were not (McNaught *et al*., 2003). These studies provide more examples of how dysfunction in protein proteolysis can contribute to neurodegeneration.

Many neurodegenerative diseases, including PD and LBD, are marked by multiple different protein aggregates. Research has proven that there are important interactions between different amyloidogenic proteins. It was recently shown that α-synuclein can initiate tau formation and that α-synuclein and tau can then synergize one another’s polymerization, demonstrating another example of how defective proteins can have a positive feedback relationship. An additional discovery about amyloidogenic protein relationships revealed that increased levels of Aβ peptides can promote the formation of intracellular tau and α-synuclein aggregates (Giasson *et al*., 2003). However, the exact mechanism by which this process occurs remains obscure. The interactions between these proteins, be they either direct or indirect, may explain the overlap in clinical and pathological characteristics of various neurodegenerative diseases. Using three novel antibodies: T22, which recognizes tau oligomers, and Syn33 and F8H7, which recognize α-synuclein oligomers, recent imaging has demonstrated that tau oligomers and α-synuclein oligomers colocalize and appear in the same aggregates, forming hybrid oligomers. The same study observed evidence that the two interact with one another’s aggregation via a toxic synergism (Sengupta *et al*., 2015).

### Proteasome impairment and huntingtin

Huntington’s disease is a polyglutamine repeat disorder, dominantly inherited and caused by abnormal expansions of long glutamine sequences found in huntingtin (Venkatraman *et al*., 2004). The disease causes motor deficits and cognitive disturbances, leading to progressive dementia and eventually death 15–20 years after disease onset. The pathology of HD, like many neurodegenerative disorders, involves the existence of intracellular and extracellular protein aggregates (Landles & Bates, 2004).

Huntingtin has been found to be ubiquitinated, suggesting that it is meant to be degraded by the UPS (Kalchman *et al*., 1996). Additionally, elevated levels of ubiquitin-reactive neurites have been observed in HD brains (Cammarata *et al*., 1993). Small ubiquitin-like modifier has been shown to be attached at lysines in the N terminus of huntingtin very near the polyglutamine stretch (Ross & Poirier, 2004). A recent study found that a pathogenic fragment of huntingtin (Httex1p) can be modified either by SUMO-1 or by ubiquitin on identical lysine residues. In cultured cells, sumoylation stabilized Httex1p, reduced its ability to form aggregates, and promoted its capacity to repress transcription. In a Drosophila model of HD, sumoylation of Httex1p exacerbated neurodegeneration, whereas ubiquitination of Httex1p abrogated neurodegeneration. Therefore, it appears that in certain situations, the two systems may lead to opposing downstream effects (Steffan *et al*., 2004). These studies provide further evidence that covalent modification and the proteasome are key components of neurodegeneration.

### Proteasome impairment and amyloid-β

Alzheimer’s disease is recognized as the most prevalent cause of late-life cognitive impairment in humans. It is a progressive form of dementia without any effective treatment (Selkoe, 2013). The accumulation of aggregated Aβ protein into insoluble fibrillar deposits known as plaques is one of the main hallmarks of AD (Terry, 2001) and is linked to UPS dysfunction (de Vrij *et al*., 2004). Studies have also reported elevated levels of ubiquitin in the cerebrospinal fluid of patients with AD (Kudo *et al*., 1994). The relationship between AD and the UPS has been investigated and continues to be an area of great research interest.

One study clearly demonstrated a correlation between proteasome inhibition and the accumulation of detergent-soluble ubiquitinated (SUb) proteins, a critical early event in the process leading to neuronal death (Metcalfe *et al*., 2012). The data collected suggest that a boost in proteasome activity is a potential mechanism for delaying or preventing toxicity. Additionally, a mutant form of ubiquitin, termed Ub^+1^, is selectively observed in the brains of patients with Alzheimer’s, including those with nonfamilial AD (Lam *et al*., 2000). The proteasome system in aging brains is especially vulnerable to this expression. Several cortical areas in patients with AD were found to have decreased levels of proteasome activity (de Vrij *et al*., 2004). All of this evidence suggests that UPS impairment plays a role in AD progression.

The research surrounding Aβ plaques is complex in that they have been linked to neurotoxicity but have not been definitively and unanimously determined to be the true origin of the disease. Some evidence suggests that the plaques are actually neuroprotective. The plaques have, in fact, been observed in individuals not affected with AD (Katzman *et al*., 1988).

Aβ is dangerous to neural health, not only because it forms toxic aggregates, but also because of the interactions it can have with other components of the cell. A recent study found that as AD advances, monomeric and oligomeric Aβ interactions with phosphorylated tau in neurons increase. Furthermore, the results suggest that these interactions damage neuronal structure and function, particularly in synapses, and thus speed the loss of cognitive function (Manczak & Reddy, 2013). Thus, tau and Aβ proteins in AD appear to utilize a positive feedback mechanism. As one accumulates, it speeds the accumulation of the other. This is just one example of toxic proteins exhibiting a tendency toward synergism with one another. Understanding how and why these interactions occur is currently the subject of extensive research.

Soluble Aβ aggregates called oligomers have recently been suggested as the true toxic species (Haass & Selkoe, 2007), representing an intermediate in the early formation of larger deposits (Hoshi *et al*., 2003). Many studies have shown that Aβ oligomers are more toxic than the more mature fibrils, and therefore, the oligomers have gained acceptance as the most toxic species in AD (Kayed & Lasagna-Reeves, 2013; Guerrero-Munoz *et al*., 2014). Moreover, Aβ oligomers can induce endoplasmic reticulum stress, leading to increased ubiquitin levels (Umeda *et al*., 2011), and soluble Aβ has been shown to directly inhibit UPS function (Almeida *et al*., 2006; Park *et al*., 2009). Aβ protein is thought to acquire its damaging characteristics early on in its formation, suggesting that UPS function is failing to degrade the harmful protein early enough to prevent toxicity (Hoshi *et al*., 2003).

### Proteasome impairment and Tau protein

Tau is a uniquely ubiquitous protein implicated in the majority of neurodegenerative diseases as a secondary amyloidosis, as well as present alone in a number of pure tau pathology disorders. Parkinson’s disease is characterized by tau protein aggregation in addition to α-synuclein. Brains affected with PD are not only full of Lewy bodies and Lewy neurites, but also of NFTs composed of accumulated tau (Lei *et al*., 2010). Additionally, tau protein has been found in the filaments that make up Lewy bodies (Arima *et al*., 1999). As mentioned earlier, tau protein also shows a tendency to co-localize and interact with α-synuclein in such a way that the two proteins bolster each other’s toxicity. As with most neurodegenerative diseases, it is not known exactly how tau contributes to PD. However, it is clear that tau is a key protein implicated in PD and its importance is becoming increasingly more evident as more research is completed.

While tau has been known to be involved in AD for many years, recently researchers have begun to view it as a critical mediator of toxicity in disease, setting aside the old paradigm in which extracellular Aβ plaques were considered the most important therapeutic target. Alzheimer’s disease is characterized by intracellular NFTs comprised of tau proteins (Takahashi *et al*., 2008) as well as tau oligomers (Lasagna-Reeves *et al*., 2012b). While multiple studies have reported ubiquitin accumulation in the NFTs found in AD (de Vrij *et al*., 2004), the relationship between the late-stage metastable fibrillar aggregates of tau and ubiquitin is complex. Neurofibrillary tangles are composed of paired helical filament (PHF) tau in a largely hyperphosphorylated state. However, studies suggest that the interaction between ubiquitin and tau is not entirely dependent on the phosphorylation state of tau. An early study investigating different fractions of tau aggregates found that ubiquitination appeared to associate with tau aggregates late in disease (Köpke *et al*., 1993). While phosphorylated and non-phosphorylated soluble tau isolated from AD brain was not ubiquitinated, phosphorylated insoluble PHFs were associated with ubiquitin (Köpke *et al*., 1993). Moreover, decreased proteasome activity in AD brains does not correlate with levels of phosphorylated tau (Keck *et al*., 2003). Independent of phosphorylation status, PHFs were also found to directly inhibit the activity of proteosomes and were associated with impaired proteosomes isolated from the disease (Keck *et al*., 2003). Tau has been implicated in HD as well; however, it does not appear to interact with huntingtin in the disease (Fernandez-Nogales *et al*., 2014). The role of tau in HD is less obvious than its role in other disorders, but the limited data available show that it may be of great importance and is worthy of further investigation. Total tau levels are higher in HD brains than in healthy brains, and the neuronal nuclei in HD brains are characterized by rod-like tau deposits, termed “nuclear rods” (Fernandez-Nogales *et al*., 2014). Furthermore, one study found that total tau levels in the cerebrospinal fluid of patients with HD were higher than in healthy patients (Constantinescu *et al*., 2011).

While mutations in tau have not been shown to be associated with AD, PD, or HD, other tauopathies, such as frontotemporal lobar dementia, can be caused by a mutation in the gene encoding tau protein (Ross & Poirier, 2004). Tau is a common histopathological hallmark of various other neurodegenerative diseases, as well as traumatic brain injury (Gerson *et al*., 2014a). Research has shown that tau aggregation and degradation have a clear, albeit complex and multifaceted connection with UPS dysfunction (Lee *et al*., 2013).

## Tau oligomers as the toxic species in disease

Tau undergoes many post-translational modifications including glycosylation, ubiquitination, glycation, polyamination, nitrosylation, truncation, and phosphorylation (Alonso *et al*., 2008). Hyperphosphorylation is thought to be the most important and disease-relevant tau post-translational modification, as it can alter tau’s biological functions and enhance tau self-assembly, aggregation, and accumulation (Avila, 2000; Lee *et al*., 2001). Hyperphosphorylation decreases tau’s affinity for microtubules and thus increases the amount of free-floating tau in the cytoplasm. As a consequence, tau may form oligomers and NFTs which correlate with neural loss, cognitive decline, synaptic deficits, protein synthesis impairment, and sequestration of transcription factors (Bretteville & Planel, 2008; Metcalfe *et al*., 2012). Recently acquired data have shown that soluble prefilamentous forms of tau may be the most toxic and pathologically significant tau aggregates (Marx, 2007; Brunden *et al*., 2008; Gerson *et al*., 2014a). Studies on living brains with tau pathology suggest that neurons undergo a slow nonapoptotic form of cell death in tauopathies (Spires-Jones *et al*., 2009).

There is controversy in the field about which tau species, whether it be monomeric tau, hyperphosphorylated tau, or oligomeric tau, causes neurotoxicity (Berger *et al*., 2007; Brunden *et al*., 2008). In fact, some studies suggest that tau may be neuroprotective (Ubhi *et al*., 2009). However, the vast majority of research shows strong evidence that tau causes neurotoxicity, even if the specific mechanism by which tau exerts this neurotoxicity is not known. Neurofibrillary tangles have long been accepted as a pathological hallmark of various tauopathies, notably AD. Although, the exact mechanism of pathological tau spreading is unclear, a growing body of research supports the hypothesis that the intermediate form of tau between monomers and NFTs known as tau oligomers is the origin of toxicity in the disease and therefore the best therapeutic target (Gerson & Kayed, 2013b).

Tau oligomers have exhibited the ability to self-propagate and to enter and exit cells, spreading from affected areas to unaffected areas of the brain (Walker *et al*., 2013). Additionally, a study on aged mice expressing human tau concluded that there was no correlation between tau filaments and cell death, suggesting that the final form of tau is not as pathologically significant as once thought (Andorfer *et al*., 2003). Extensive research is devoted to developing cellular assays that can be used to target tau oligomers (Davidowitz *et al*., 2008). In fact, a tau oligomer-specific antibody, T22, has recently been engineered in our laboratory and used in a study that supported the premise of oligomers as therapeutic targets in tauopathies (Lasagna-Reeves *et al*., 2012a). The development of such antibodies not only reveals causes of neurotoxicity but also the potential of immunotherapies as a counteraction to the progression of the disease.

We also recently engineered a novel anti-tau oligomer-specific mouse monoclonal antibody (TOMA). This antibody was successfully used for passive immunotherapy in two different tauopathy mouse models. The results showed that long-term administration of TOMA was effective as a preventative therapy, inhibiting oligomeric tau and preserving memory and motor function. These data support the critical role of oligomeric tau in disease progression and validate tau oligomers as a potential drug target (Castillo-Carranza *et al*., 2013, 2014a,b).

### The harmful effects of tau oligomers on the UPS

In relation to the UPS, these findings suggest that an early error in the process of tau development can hinder proper proteolysis. Further supporting this hypothesis is a recent study that found a correlation between the accumulation of hyperphosphorylated tau oligomers at human AD synapses, increased ubiquitinated substrates, and increased proteasome components, consistent with dysfunction of the UPS (Tai *et al*., 2012).

If faulty tau proteins could be destroyed before causing harm, the death of cells could potentially be avoided. It is not uncommon for the cell to make errors during protein synthesis. However, in an ideal cellular environment, the faulty proteins that are a result of the said mistakes are broken down and are not afforded an opportunity to harm the cell. However, in neurodegeneration, it appears that the proteasome is unable to digest these unwanted proteins. As a result, the proteins accumulate and presumably cause more proteasome impairment. Therefore, ubiquitination and sumoylation have a role in the process of tau becoming neurotoxic because they have the capability of destroying tau before it can harm the cell.

### Ubiquitination of tau oligomers

A widely recognized indication of a relationship between the UPS and toxic tau is that the tau aggregates found in degenerating neurons are not only phosphorylated, but are also ubiquitinated (de Vrij *et al*., 2004). To investigate this relationship in greater detail, one study used neuroblastoma cells overexpressing tau proteins and showed, surprisingly, that proteasome inhibition led to a bidirectional degradation of tau (Delobel *et al*., 2005). One explanation of these results is that when the proteasome is inhibited, the cell compensates by overexpressing caspases or calpain, both proteases capable of proteolyzing tau. This phenomenon could explain why caspase activation seems to correlate with the degradation of tau and why calpain has been shown to be overactivated in the neurodegenerative process (Delobel *et al*., 2005). This evidence demonstrates a clear yet very complex relationship between tau and the UPS. While there is certainly a connection between tau aggregates and UPS impairment, how exactly this connection is forged needs to be investigated further in order to be fully understood. It appears that overcompensation with proteases such as caspase and calpain cannot fully clear the defective tau out of the cell, at least in some cases, as with neurodegenerative disorders.

### Sumoylation of tau oligomers

Many proteins involved in neurodegeneration are sumoylated, including tau, which is also established to be regulated by phosphorylation. Phosphorylation has been shown to decrease the affinity of tau to the microtubules, upon which it may become unstable and aggregate (Biernat *et al*., 1993; Bramblett *et al*., 1993; Sengupta *et al*., 1998). Freeing tau from its site on the microtubules may also allow aggregates to interact with the endosomal/lysosomal system or alter the permeability of the cell membrane, thereby allowing it to be released into the extracellular space where it may contribute to the spread of pathology (Gerson & Kayed, 2013a; Holmes & Diamond, 2014). Treatment with the phosphatase inhibitor, okadaic acid, or the microtubule depolymerizing drug, colchicine, up-regulated tau sumoylation. This suggests that SUMO modification may preferentially target a free soluble pool of the substrate (Dorval & Fraser, 2006).

A recent study on transgenic mice expressing Aβ precursor protein and mutant tau found that SUMO-1 protein was co-localized with phosphorylated tau coexisting with Aβ in plaques (Takahashi *et al*., 2008). Evidence for the potential importance of SUMO modifications in tau processing is further supported by the fact that sumoylation of tau is stimulated by phosphorylation, a post-translational modification known to be associated with tau in disease states (Dorval & Fraser, 2006).

The sumoylation of tau oligomers is at the center of upcoming research projects for a good reason: It has many implications with the UPS and the potential to lend insight into the relationship between oligomers and the proteasome.

## Therapeutic strategies

There are many defensive cellular mechanisms against protein aggregation and the misfolding that precedes the said aggregation, and accordingly many points at which these defensive pathways can fail. Therefore, there are numerous steps in the process of protein formation and in various other cell processes that are worthy of investigation as potential therapies against neurodegeneration. The first line of defense includes the chaperones that aid in proper protein folding and refolding of abnormalities (McClellan & Frydman, 2001). Second in line is the option of covalent modification, as with ubiquitin or sumoylation, followed by proteasome digestion (Goldberg, 2003). The fact that the pathological inclusions of neurodegenerative diseases contain many of the components necessary for the cell’s protection against these same inclusions, notably chaperones, ubiquitin, and proteasome constituents, presumably represents cellular defenses overwhelmed by the excessive aggregation within cells (Ross & Poirier, 2004). Many pharmaceutical companies are investing in the targeting of the UPS, not only for neurodegenerative disease, but also for cancer and various other disorders. However, proteasome inhibition under investigation as a potential therapeutic for cancer must be reviewed with caution in light of potential effects for neurodegeneration. The dipeptide boronic acid analog bortezomib, commercially dubbed Velcade™, recently became the first proteasome inhibitor to reach human trials (Adams & Kauffman, 2004). Bortezomib inhibits the proteasome itself and leads to the destruction of cancer cells via a build-up of toxic proteins. Although the lack of specificity of this inhibiting action causes multiple unwanted side effects, cancer cells appear to be more sensitive to the effects of proteasome inhibition than normal cells due to a loss of checkpoint mechanisms occurring during tumorigenesis. Normal cells can usually recover as the proteasome inhibition is transient and reversible (Field-Smith *et al*., 2006). It is unknown how such a treatment would affect amyloid proteins in the brain. Researchers are also investigating the efficacy of treating with sumoylation inhibitors (Chen & Li, 2012).

## Conclusion

The covalent modification of tau, amyloid-β, and α-synuclein oligomers clearly has an impact on neurotoxicity. Ubiquitin and SUMO, two tags thought to target molecules for degradation, exist in the toxic protein aggregates that characterize neurodegenerative diseases, suggesting that while the cell does not have trouble recognizing harmful proteins, the proteasome may have trouble breaking them down. This could be because the toxic species have certain mechanisms to protect themselves against digestion, which would in part explain why so few neurodegenerative diseases have effective treatments or cures.

**Fig 1:**
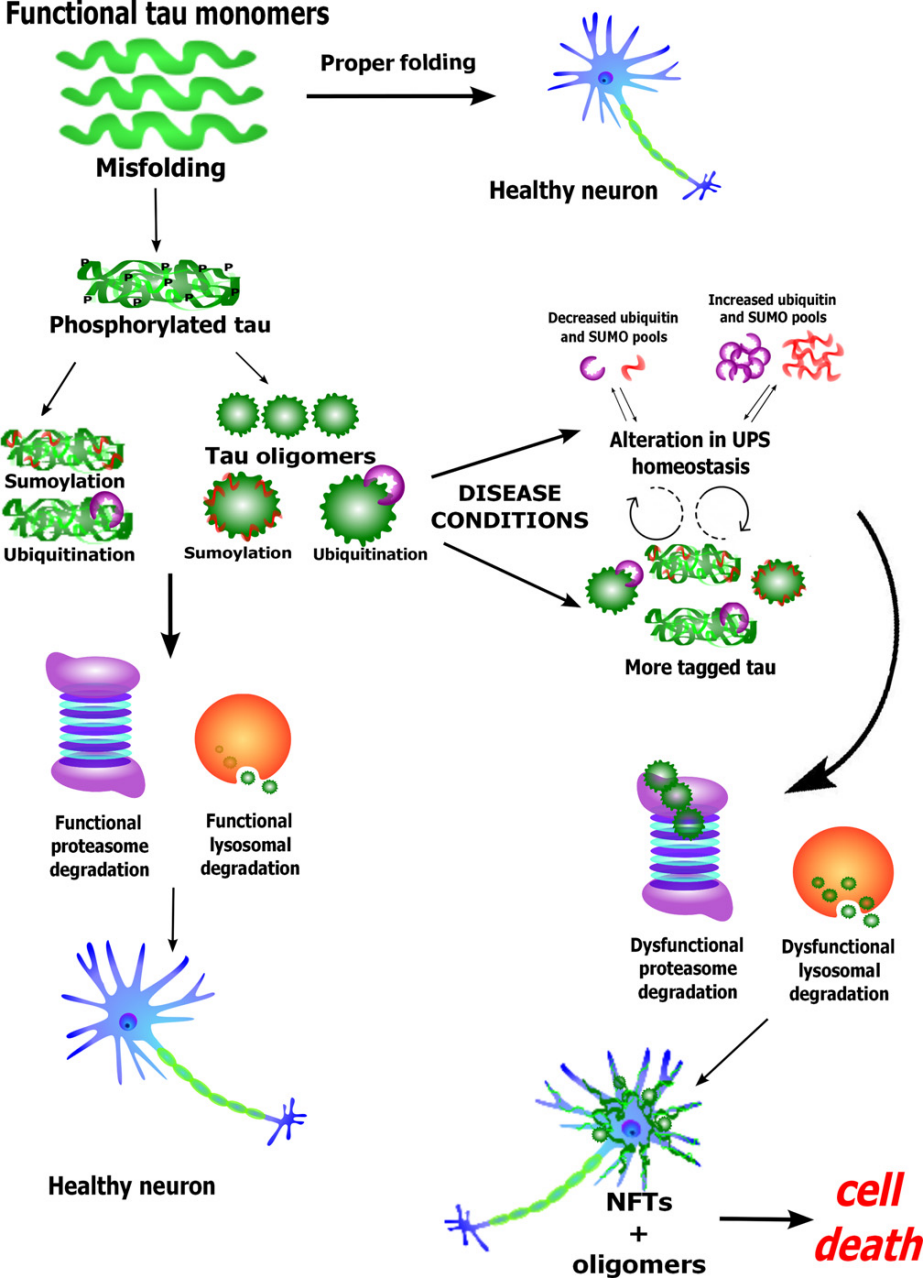
Schematic depicting a hypothesis of how errors in the proteolytic pathway can lead to cell death.

One of the most challenging parts of combing through the abundant and often contradictory data surrounding the subject of proteolysis in neurodegenerative diseases is differentiating between causation and correlation. Amyloid protein accumulation has for many years been recognized as a cause of neurotoxicity, but experimentation and critical examination demonstrate that the seeds of neurotoxicity develop earlier than previously thought in the form of oligomers. These discoveries suggest that accumulation of fibrillar aggregates is an outcome rather than a cause of toxicity. However, smaller aggregates are still undoubtedly toxic and their damaging effects on the cell appear to exacerbate the cause of toxicity, resulting in a snowball effect which explains why neurodegeneration advances at an increased rate the longer it exists in the brain. If the cell was able to recognize harmful molecules and destroy them before they became dangerous, as a healthy cell is presumably able to do, neurotoxicity could be avoided altogether. Brains afflicted with diseases such as Alzheimer’s, Parkinson’s, Huntington’s, and others appear to have impaired proteolytic pathways, allowing these harmful proteins to build up to toxic levels. This possible mechanism is summarized in Fig.1.

The ambiguity of causation versus correlation is especially evident in the question of whether or not ubiquitination and sumoylation themselves are a source of toxicity. At face value, it may seem to be the case because of the ample concurrence of covalent modification and neurodegeneration. Critical examination of the cell’s systems and analysis of previous observations lend a hypothesis of how this tagging could cause proteasome impairment and thus toxicity in a roundabout manner. If the misfolded proteins were not tagged with ubiquitin or with SUMO, they would not be sent to the proteasome. Instead, they may be completely digested by cellular mechanisms such as lysosomes and would not aggregate. Therefore, it is possible that without heightened ubiquitination and sumoylation, the proteasome would not become congested and would not have trouble degrading misfolded proteins. A functional proteasome may be able to prevent aggregates from ever forming in the first place. In this way, the tagging itself is a cause of disease, albeit indirect. Nevertheless, the real issue seems to be the impairment of the proteasome itself. Thus far, no one has thoroughly investigated the direct impact of tau oligomers on the UPS. However, evidence from other amyloidogenic protein oligomers, as well as correlative evidence about the relationship between tau and ubiquitin, suggests that tau oligomers may lead to impairment of the proteasome, which is worthy of further investigation. This line of research may prove to be crucial to discovering the mechanism and potential treatments for neurodegeneration in the future.
